# Wavelength-Tunable L-Band High Repetition Rate Erbium-Doped Fiber Laser Based on Dissipative Four-Wave Mixing

**DOI:** 10.3390/s21175975

**Published:** 2021-09-06

**Authors:** Kai Li, Qianqian Huang, Junjie Jiang, Zinan Huang, Chengbo Mou

**Affiliations:** Key Laboratory of Specialty Fiber Optics and Optical Access Networks, Joint International Research Laboratory of Specialty Fiber Optics and Advanced Communication, Shanghai Institute for Advanced Communication and Data Science, Shanghai University, Shanghai 200444, China; li_kai@shu.edu.cn (K.L.); huangqq@shu.edu.cn (Q.H.); jiangjunjie@shu.edu.cn (J.J.); zinanhuang@shu.edu.cn (Z.H.)

**Keywords:** dissipative four-wave mixing, wavelength tunability, high repetition rate, erbium-doped fiber laser

## Abstract

A wavelength-tunable high repetition rate (HRR) erbium-doped fiber laser in L-band based on dissipative four-wave mixing (DFWM) mechanism is demonstrated. The cavity can generate a single-soliton train and bound-soliton train with a fixed repetition rate of ~126 GHz, which is determined by the free spectral range of the intra-cavity Lyot filter. A wide wavelength-tuning operation can also be obtained by rotating the polarization controllers. The wavelength-tuning ranges of the HRR single-soliton state and HRR bound-soliton state are ~38.3 nm and ~22.6 nm, respectively. This laser provides useful references for the area of a wavelength-tunable fiber laser with high repetition rate. The laser may also find useful applications in high-speed communication, sensing, etc.

## 1. Introduction

In recent decades, mode-locked fiber lasers with wavelength tunability have attracted great attention for various applications, such as micromachining, sensing, spectroscopy, and optical communication [1,2,3]. In particular, for high-speed optical communication systems, the conventional C-band (1530-1565 nm) is unable to meet the increasing requirement of communication capacity. Therefore, it is of great significance to investigate L-band (1565–1625 nm) wavelength-tunable mode-locked fiber lasers. Experimentally, there are two major ways to achieve wavelength tunability. The first is incorporating a tunable spectral filter in the cavity, such as a W-shaped long-period fiber grating (LPFG) filter [4], chirped fiber Bragg grating [5], birefringence-induced fiber filter [6], and commercial tunable band-pass filter [7]. The other method is to control intra-cavity loss by using some special elements, such as a tunable-ratio optical coupler [8], fiber taper [9], mechanical attenuator [10], and LPFG-based narrow-band optical attenuator [11]. However, these conventional wavelength-tunable EDFLs mostly operate at the repetition rate of megahertz. In order to make full use of the L-band for optical communication, it is also necessary to improve the repetition rate of the EDFLs to gigahertz (GHz).

To achieve a high-repetition rate (HRR) pulse train, a variety of passive mode-locking mechanisms have been reported, such as harmonic mode locking [12,13,14], short cavity [15,16], and dissipative four-wave mixing (DFWM) [17,18]. Compared to harmonic mode locking and short cavity, DFWM is a simpler and more effective method to generate an HRR pulse train with a capability even up to terahertz. In order to achieve a DFWM mode-locking operation, large nonlinearity is necessary, which contributes to phase matching between longitudinal modes in the cavity. Such intra-cavity nonlinearity can be enhanced by inserting a segment of highly nonlinear fiber, i.e., photonic crystal fiber [19]. In addition, an intra-cavity comb filter is mandatory. Due to the unflatness of gain spectrum and limited gain bandwidth of gain fiber, a two-hump filtering configuration is formed by a combination of a comb filter and a segment of gain fiber. Therefore, under the joint function of this two-hump filtering effect and intra-cavity nonlinearity, only two modes selected by this two-hump filtering effect can experience net gain and transfer their energy into high-order harmonics unidirectionally, which are in the region of negative gain by a four-wave mixing effect. Then, an HRR pulse train is finally formed with a repetition rate defined by the free spectral range (FSR) of the comb filter. During the past decades, various schemes based on DFWM have been reported, such as sampled fiber Bragg grating [18,20,21,22], F-P filters [23,24], Mach–Zehnder interferometers [25,26], Lyot filters [27,28], programmable optical processors [29,30], and microring resonators [31,32]. In particular, J. Schröder et al. experimentally obtained up to a 20 nm wavelength tunability by incorporating a programmable optical processor in the cavity [29]. However, the large insertion loss and cost of the programmable optical processor make it impossible to be widely used. Through inserting an F-P filter, X. M. Tan et al. achieved a wavelength-tunable and switchable dual-waveband operation in an HRR EDFL, while the wavelength-tuning range was less than 15.2 nm [33]. Considering the importance of L-band and wavelength tunability, it is interesting to investigate a widely wavelength-tunable L-band HRR EDFL.

Herein, we experimentally demonstrate a widely wavelength-tunable L-band HRR EDFL for the first time. A pulse train with a repetition rate of ~126 GHz is obtained. In our laser, a fiber-based Lyot filter composed of a polarization dependent isolator (PD-ISO) and a piece of polarization-maintaining (PM) fiber is used to achieve a DFWM effect. Due to the existence of such a Lyot filter, the cavity can generate two kinds of HRR pulse trains. One is a single-soliton HRR pulse train with a wavelength-tuning range of ~38.3 nm (from 1569.83 nm to 1608.21 nm). The other status is an HRR bound-soliton pulse train, which can be achieved by rotating polarization controllers (PCs). In this state, the spectral center wavelength can be adjusted from 1578.98 nm to 1601.60 nm (~22.6 nm). Moreover, the conventional noise-like pulse (NLP) can also be observed when the intra-cavity polarization state is adjusted in some specific regime. We believe this fiber laser will provide an important reference for future research on wavelength-tunable HRR EDFLs.

## 2. Experimental Setup

**Figure 1:**
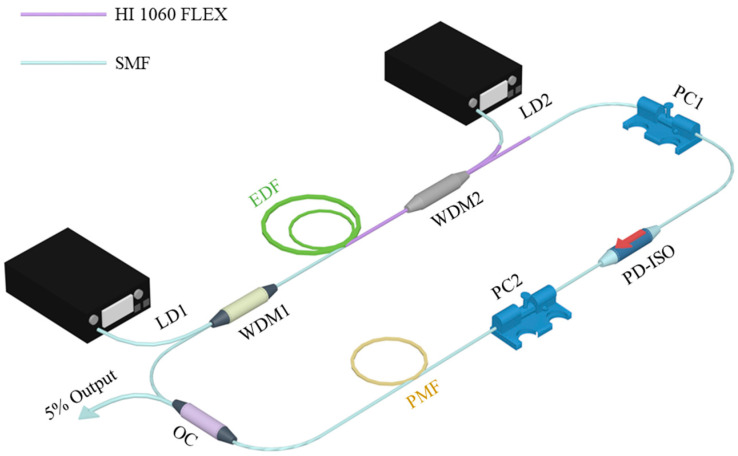
Experimental setup of HRR erbium-doped fiber laser.

The experimental setup of the fiber laser is shown schematically in Figure 1. The total cavity length is ~12.53 m, composed of a 2.59 m long erbium-doped fiber (EDF) (OFS Er80) with a group velocity dispersion (GVD) of +61.2 ps^2^/km as the gain medium, 1.23 m HI 1060 FLEX with a GVD of −7 ps^2^/km, 5.51 m PM fiber (YOFC, PM1550 125-13/250) with a GVD of −22.8 ps^2^/km, and ~3.20 m single mode fiber (SMF) with a GVD of −22.8 ps^2^/km. The net cavity dispersion is ~−0.048 ps^2^. Two 980 nm laser diodes (LDs) provide a total maximum pump power of ~1.66 W (LD1, CM97-1050-76 with maximum power is ~1W and LD2, YMPSS-980-660-B-FBG with maximum power is ~660 mW) from two directions, through two wavelength division multiplexers (WDM). A PD-ISO is inserted into the cavity to ensure the light is linearly polarized and to prevent bidirectional transmission. Two PCs are used to adjust intra-cavity polarization, and the 5:95 optical coupler (OC) outputs 5% laser energy.

Due to the existence of PD-ISO and PMF, a wavelength-dependent birefringence-induced filter with a comb transmission spectrum is formed in the cavity [34,35]. This filter is also called a Lyot filter and its transmission function can be described as [36,37]

(1) $$T=\cos^{2}\theta_{1}\cos^{2}\theta_{2}+\sin^{2}\theta_{1}\sin^{2}\theta_{2}+\frac{1}{2}\sin\left(2\theta_{1}\right)\sin\left(2\theta_{2}\right)\cos\left(\Delta \varphi_{L}+\Delta \varphi_{\mathrm{NL}}\right)$$

In Equation (1), $\theta_{1}$ and $\theta_{2}$ are azimuth angles of the two ends of the polarizer relative to the fast axis of the PMF. $\Delta \varphi_{L}=2\pi L\Delta n/\lambda$ is the linear phase delay, and $\Delta \varphi_{\mathrm{NL}}=2\pi n_{2}PL\cos\left(2\theta_{1}\right)/\left(\lambda A_{eff}\right)$ is the nonlinear phase delay. L and Δn are length and birefringence intensity of PMF, respectively. λ is laser’s operating wavelength, $n_{2}$ is nonlinear coefficient, P is instantaneous power of optical signal, and $A_{eff}$ is effective area of core of PMF. Additionally, the FSR of fiber-based Lyot filter can be calculated by [27]

(2) $$\Delta \lambda =\frac{\lambda^{2}}{L\Delta n}$$

**Figure 2:**
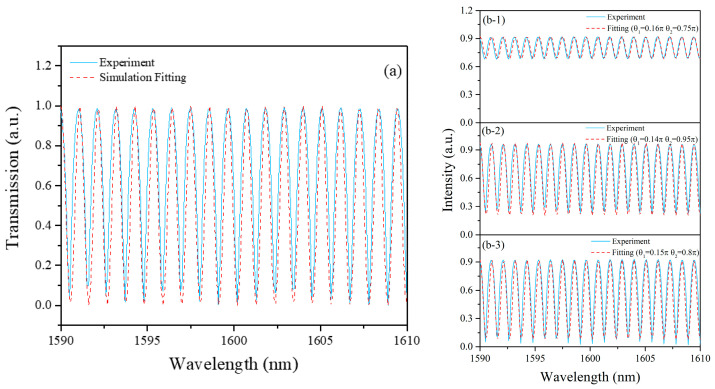
(**a**) Transmission spectrum of fiber-based Lyot filter in L-band (solid, experimental result; dashed, simulated result). (**b-1**–**b-3**) Transmission spectrum with different angles of $\theta_{1}$ and $\theta_{2}$ (solid, experimental result; dashed, simulated result).

The modal birefringence of PMF used in cavity is ${4.32\times 10}^{-4}$. Since the transmission spectrum of the filter is only related to PMF, we disconnected the connection between the coupler and the PMF and spliced a polarizer after the PMF to measure the transmission spectrum of the fiber-based Lyot fiber. As shown in Figure 2a, the solid line is the experimental measurement result, and the FSR is ~126 GHz. The dashed line is the simulated result, which is in good agreement with the measured results. We also measured the transmission spectrum at different polarization states, which is illustrated in Figure 2b. The depth of the transmission spectrum changes with the adjustment of the orientations of PCs. We also simulated the transmission spectrum under different angles of $\theta_{1}$ and $\theta_{2}$ (dashed line in Figure 2(b-1,b-2)), which is in good agreement with the experimental results.

## 3. Experimental Results and Discussions

### 3.1. HRR Output with the Repetition Rate of ~126 GHz

**Figure 3:**
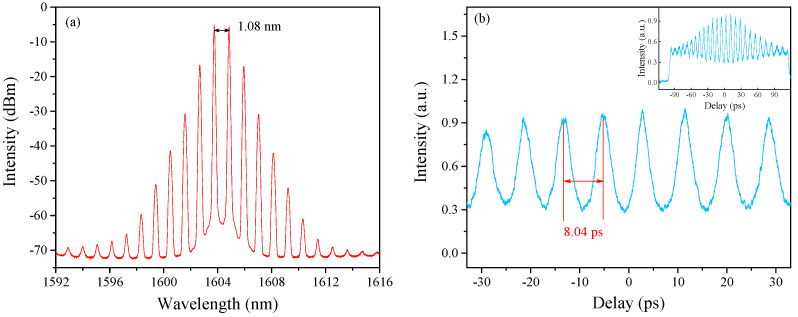
Output characteristics of laser cavity based on Lyot filter-driven DFWM. (**a**) Optical spectrum. (**b**) AC traces; the inset figure is the full scanning measured result.

Due to the spectral filtering effect of the fiber-based Lyot filter, the DFWM mode-locking operation self-starts under the forward (LD1) and backward (LD2) pump power of 332 mW and 200 mW. Figure 3 shows a stable HRR single-soliton train when the pump power of LD1 and LD2 is 865 mW and 500 mW. The output power of the cavity is 13.69 mW. Figure 3a is the measured comb spectrum with a center wavelength of 1604.30 nm and a peak-to-peak interval of 1.08 nm, which corresponds to ~126 GHz. Here, we attribute this L-band oscillation to the in-band absorption of EDF [38,39]. The autocorrelation (AC) trace of the single-soliton train shown in Figure 3b has a pulse-to-pulse interval of 8.04 ps, which is in good agreement with the interval of spectral peaks. Additionally, the inset in Figure 3b is the measurement result across the entire scanning range. It is noted that the AC traces have a continuous wave background, and the pulse sequence is not flat. We speculate that this unflatness of the pulse intensity may be caused by the modulation instability of the two main spectral peaks [27].

**Figure 4:**
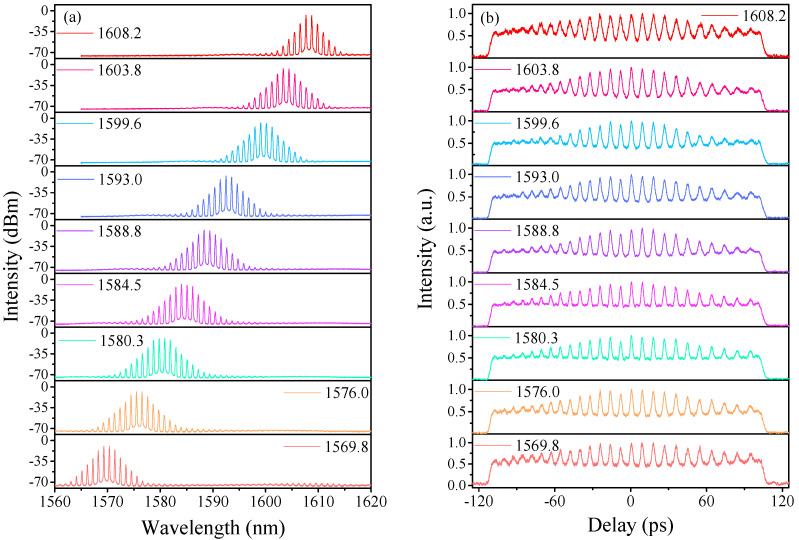
HRR single-soliton train for various orientations of PCs. (**a**) Optical spectra at various central wavelengths. (**b**) Corresponding AC traces.

When we keep the pump power of LD1 and LD2 fixed at 715 mW and 500 mW, the wavelength tuning operation can been achieved by carefully adjusting PCs. As illustrated in Figure 4a, the center wavelength of the HRR single-soliton train can be tuned from 1569.83 nm to 1608.21 nm, with a wavelength tuning range of ~38.3 nm in the L-band. To our best knowledge, this is the widest spectral-tuning range from which the DFWM fiber laser works in the L-band so far. During the process of wavelength tuning, the HRR single-soliton train has been well maintained, which is demonstrated in Figure 4b. We believed that such wavelength tunability is attributed to broad transmission characteristics of the intra-cavity Lyot filter in the L-band. The orientations of PCs lead to the change of modulation depth of the Lyot filter and the position of maximum gain, which shift the center wavelength of output pulses. In particular, this spectral tuning process is reversible.

**Figure 5:**
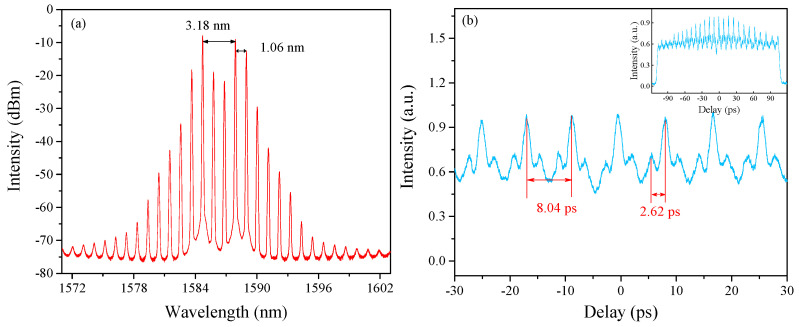
HRR Bound-soliton train. (**a**) Optical spectrum. (**b**) AC traces; the inset figure is full span measurement.

Keeping the pump power fixed and further adjusting the orientations of PCs, the bound-soliton state can be obtained with the output power of 8.275 mW and the repetition rate of a unit of bound solitons also matches the frequency spacing of the Lyot filter. We believe that modulation instability has played a role in the formation of the HRR bound-soliton train, altering the repetition unit of the cavity emission [27]. The measured results are shown in Figure 5. The middle of the spectrum is concave, and the peaks on two sides are the strongest in Figure 5a, which is a typical characteristic of the bound-soliton state. The peak-to-peak interval of the spectrum is 1.06 nm, corresponding to a repetition rate of ~126 GHz. Figure 5b shows the AC traces with an interval of ~8.03 ps, which is consistent with the result in the HRR single-soliton state. In addition, it seems that the pulse-to-pulse interval in a single bound soliton is related to the spectrum. The interval between two highest peaks is 3.18 nm, corresponding to a repetition rate of ~380 GHz, which matches well with the side-pulse interval of 2.62 ps. 

**Figure 6:**
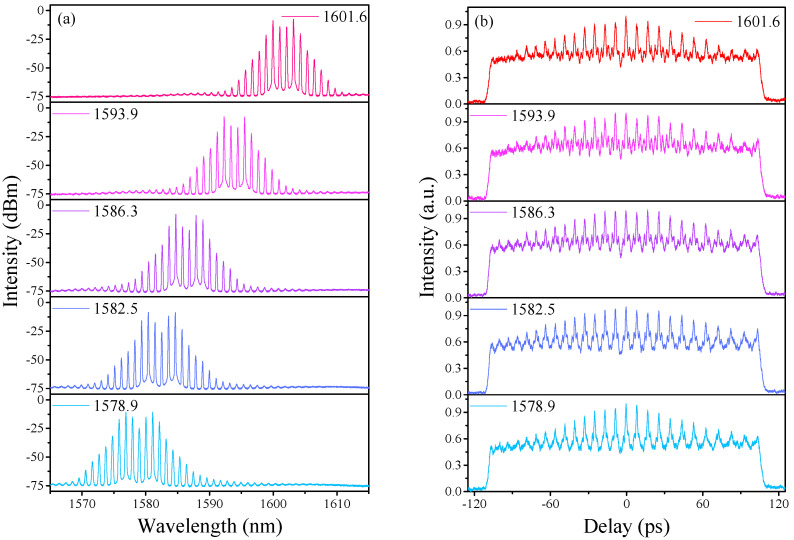
HRR bound-soliton train for different polarization states. (**a**) Optical spectra at various central wavelengths. (**b**) Corresponding AC traces.

Similar to the situation of the HRR single-soliton state, the center wavelength of the spectrum in the bound-soliton state is also tunable. As illustrated in Figure 6a, the center wavelength has been shifted from 1578.98 nm to 1601.60 nm through adjusting PCs carefully and slowly, with a wavelength tuning range of ~22.6 nm in the L-band. Figure 6b exhibits the corresponding AC traces of the HRR bound-soliton state. There always is a strong background pedestal in measured AC traces regardless of HRR single-soliton or HRR bound-soliton emissions.This phenomenon may be caused by supermode noise in the cavity [20,26] and can be suppressed by inserting a sub-cavity structure for enhanced filtering [26] or using a high-finesse filter instead [40,41,42].

### 3.2. HRR Output with the Repetition Rate of ~71 GHz

**Figure 7:**
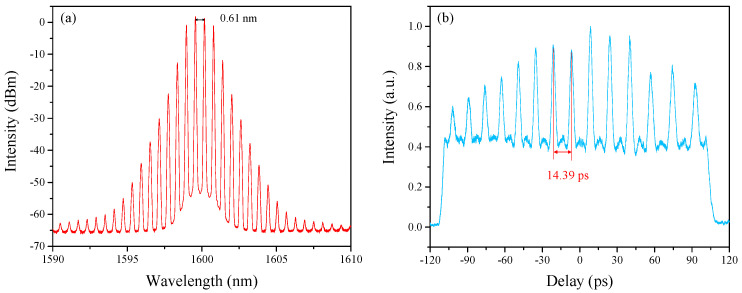
HRR single-soliton train when the length of intra-cavity PMF is 10 m. (**a**) Optical spectrum. (**b**) Corresponding AC traces.

**Figure 8:**
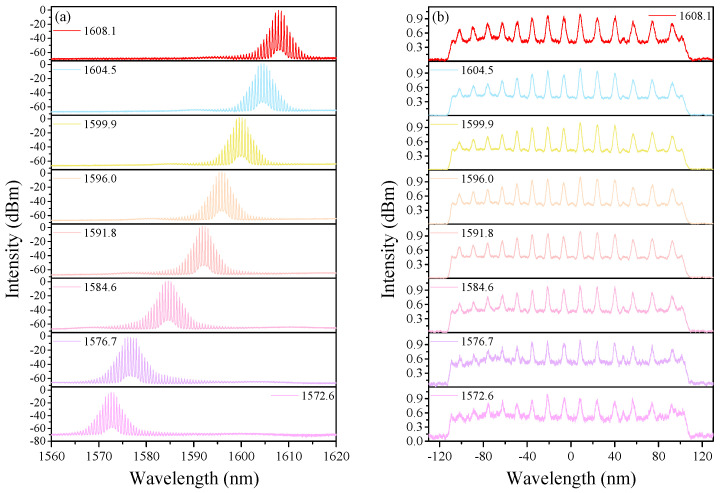
HRR single-soliton train for different polarization states when the length of intra-cavity PMF is 10 m. (**a**) Optical spectra at various central wavelengths. (**b**) Corresponding AC traces.

In our experiment, HRR EDFLs with different repetition rates can be obtained by merely changing the length of PMF. This is because the FSR of the fiber-based Lyot filter is related to the length of PMF. For example, if we want to achieve a pulse train with a repetition rate of ~70 GHz in the L-band, the calculated length of PMF is 10 m, and the corresponding FSR is ~0.6 nm. In order to verify this, we insert a 10 m PMF into the cavity. When the pump power of LD1 and LD2 is 893 mW and 660 mW, the HRR single-soliton train can be generated. Figure 7a shows the corresponding optical spectrum with a peak-to-peak interval of 0.61 nm, which is consistent with the calculated result. Additionally, the interval of pulses is measured to be ~14.39 ps. The experimental results strongly prove the efficiency of this technique. Keeping the pump power fixed, the above-mentioned wavelength-tuning operation can be obtained by adjusting PCs. As exhibited in Figure 8a, the central wavelength of the spectrum has been tuned from 1572.58 nm to 1608.12 nm, nearly 35.6 nm. Additionally, the corresponding AC traces are shown in Figure 8b. In addition, the HRR bound-soliton trains with the same repetition rate of ~71 GHz and the corresponding wavelength-tuning operation can also be achieved, which are similar to the results in Figure 5 and Figure 6.

### 3.3. Noise-like Pulse Generation

**Figure 9:**
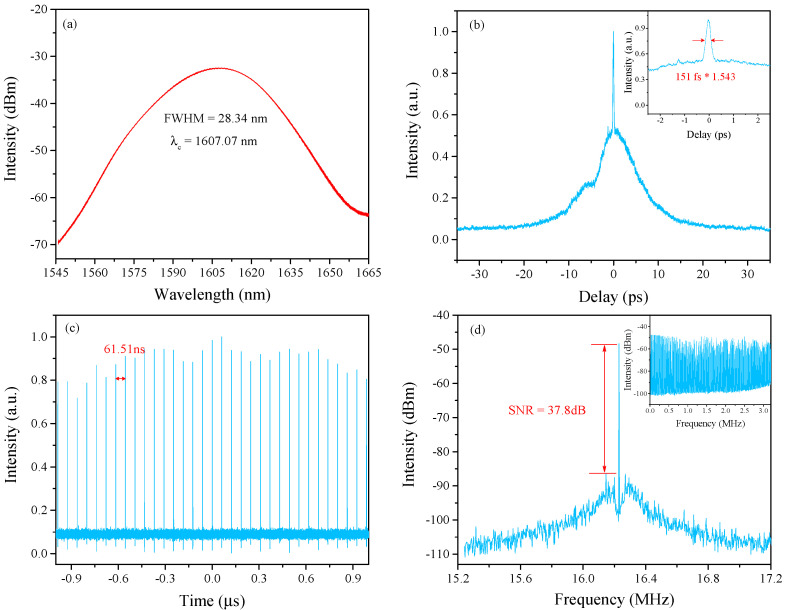
Measurement results of NLP. (**a**) Optical spectrum. (**b**) AC trace; the inset figure is the narrow spike. (**c**) Pulse train measured by oscilloscope. (**d**) RF spectrum with a span and resolution of 2 MHz and 300 Hz; the inset figure is the RF spectrum with a span and resolution of 3.2 GHz and 10 kHz.

In addition to the above-mentioned HRR pulse train emission, we can also observe NLP in this laser regardless of whether the length of the intra-cavity PMF is 5.51 m or 10 m. When the orientations of PCs were adjusted to make the transmission coefficient very small, the comb filtering characteristic of the Lyot filter was suppressed. Therefore, the DFWM effect became weak and could not support the formation of the HRR pulse train, finally resulting in the generation of NLP. For example, when the length of PMF is 5.51 m, it is observed that the NLP is generated by adjusting PCs carefully under the total pump power of 1.215 W. The measurement results are shown in Figure 9. The center wavelength and full width at half height (FWHM) of the optical spectrum are 1607.07 nm and 28.34 nm in Figure 9a. Figure 9b exhibits a typical AC trace of the NLP, which is composed of a broad pedestal and a narrow spike. Additionally, the FWHM of this narrow spike is 151 fs (sech^2^ assumed). The corresponding pulse train shown in Figure 9c is measured by the oscilloscope connecting with a high-speed photo-detector (Newport 818-BB-51F, 12.5 GHz). The pulse interval is 61.51 ns, which matches well with the cavity length. As presented in Figure 9d, the signal noise ratio (SNR) is 37.8 dB. Additionally, the RF spectrum with a 3.2 GHz span is shown in the illustration. 

### 3.4. Stability Measurements 

**Figure 10:**
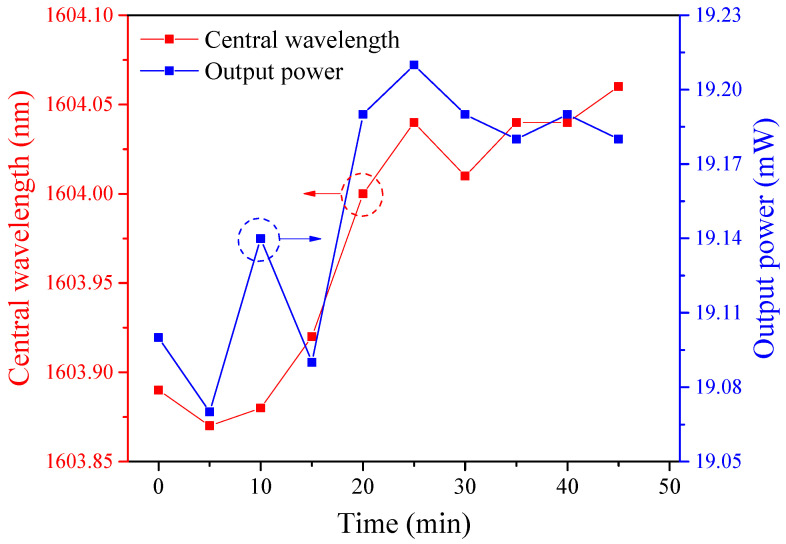
Stability measurements of central wavelength and output power for 45 min when the length of PMF is 5.51 m.

We also perform an experiment to verify the stability of this laser when the PMF is 5.51 m. Increasing the pump power of LD1 and LD2 to 893 mW and 660 mW and ensuring the cavity is working in an HRR single-soliton operation, the laser output is repeatedly recorded at an interval of 5 min for 45 min. As exhibited in Figure 10, the central wavelength drift and intensity fluctuation are 0.19 nm and 0.14 mW, respectively, indicating the good stability of this HRR EDFL.

## 4. Conclusions

In conclusion, we experimentally demonstrate a wide wavelength-tunable L-band HRR EDFL based on a DFWM mode-locking mechanism. Due to the existence of an intra-cavity fiber-based Lyot filter, the repetition rate of the pulse train is fixed at ~126 GHz. Under the forward and backward pump power of 715 mW and 500 mW, two kinds of HRR pulse sequences, i.e., HRR single-soliton train and HRR bound-soliton train, can be obtained. With the broad transmission spectrum of this comb filter, the wavelength-tuning operation has been achieved by rotating the PCs in both pulse regimes. The center wavelength-tuning range in the HRR single-soliton state and HRR bound-soliton state is ~38.3 nm and ~22.6 nm, respectively. Compared to other filters, this technique especially has better flexibility. On the one hand, this fiber-based filter allows for an all-fiber structure, which has higher compactness and lower insertion loss. On the other hand, due to the FSR of this fiber-based Lyot filter being related to the length of PMF in the cavity, we can select the appropriate length to obtain a pulse train with the required repetition rate for different applications. Our research provides an easy and effective method to achieve a wavelength-tunable GHz HRR EDFL in the L-band, which will contribute to the development of wavelength-tunable HRR EDFLs. In addition, such high-repetition-rate comb fiber lasers can also work as seed sources for sensing, such as coherent ranging [43], gas detection at 1.5–1.6 μm [44], and temperature sensing [45].

## Data Availability

Not applicable.
